# Adverse health outcomes in violent crime convicted persons: risk factors for somatic inpatient healthcare utilization

**DOI:** 10.1186/s12889-026-26983-4

**Published:** 2026-03-24

**Authors:** Joakim Jakobsson, André Tärnhäll, Eva Billstedt, Märta Wallinius, Anders Håkansson, Björn Hofvander

**Affiliations:** 1 Department of Clinical Sciences Lund, Lund Clinical Research on Externalizing and Developmental Psychopathology, Lund University, Lund, Sweden; 2 Department of Psychiatry and Neurochemistry, Centre of Ethics, Law and Mental Health, University of Gothenburg, Gothenburg, Sweden; 3 Department of Forensic Psychiatry, Region Skåne, Sweden; 4 Gillberg Neuropsychiatric Centre, Institute of Neuroscience and Physiology, University of Gothenburg, Gothenburg, Sweden; 5 Research Department, Regional Forensic Psychiatry Clinic, Växjö, Sweden; 6 Department of Clinical Sciences Lund, Faculty of Medicine, Evidence-based Forensic Psychiatry, Lund University, Lund, Sweden; 7 Department of Clinical Sciences Lund, Psychiatry, Faculty of Medicine, Lund University, Lund, Sweden; 8 Department of Forensic Psychiatry, RPC Trelleborg, Malmö, SE-205 02 Sweden

**Keywords:** Prisoners, Care continuity, Disease, Ambulatory care sensitive conditions, Injuries, Mortality, Educational status, Foster home care, Offending trajectory

## Abstract

**Background:**

Violent Crime Convicted Persons (VCCPs) are at increased risk of somatic health problems over the life course. However, the topic remains insufficiently studied, particularly regarding interactions with healthcare services in high-risk groups over extended periods. This prospective study aimed to explore adverse somatic outcomes in young adult VCCPs with a history of imprisonment and their interactions with healthcare services in Sweden.

**Methods:**

In the Development of Aggressive Antisocial Behavior Study (DAABS) cohort, male VCCPs aged 18–25 (*n* = 266) imprisoned for violent and/or ‘contact sexual offenses were clinically assessed in 2010–2012 and prospectively followed in Swedish national registries throughout 2017. Information regarding somatic inpatient healthcare utilization (HCU), somatic morbidity, and prescribed drug use was tracked and compared with a general population comparison group (*n* = 10,000). Baseline risk factors were used to explore prospective somatic inpatient HCU in VCCPs.

**Results:**

The DAABS cohort exhibited higher rates of both somatic outpatient (IRR = 1.8 [1.6–2.5]) and inpatient (IRR = 3.3 [2.2–4.9]) healthcare utilization compared with the general population group. They also showed a higher cumulative incidence of injuries of all types (IRR = 3.1 [2.4-4.0]), as well as ambulatory care sensitive conditions (ACSCs) (IRR = 2.2 [1.5–3.2]). Increased rates of prescription drug use were observed for nervous and respiratory systems, whereas reduced rates were especially noted in drugs used for the alimentary tract and metabolism as well as blood and blood forming organs. The DAABS cohort exhibited a severely elevated risk of all-cause mortality (HR 16.1 [9.4–27.8]). Low educational attainment decreased the incidence rate, while foster home placement and the assignment to a persistent offending trajectory increased the incidence rate of somatic inpatient HCU within the cohort.

**Conclusions:**

The VCCP cohort exhibited atypical patterns of somatic healthcare utilization, characterized by elevated inpatient and outpatient use, high rates of ACSCs and injuries, and a strikingly increased risk of premature mortality compared with the general population. The atypical nature of this utilization, reflected in the elevated incidence of ACSCs, underscores the need to improve understanding of the group’s HCU patterns and the potential barriers to primary care. Enhancing health literacy and reducing barriers to timely and appropriate care are essential steps toward mitigating adverse health outcomes and promoting healthcare equity in this vulnerable population.

**Supplementary Information:**

The online version contains supplementary material available at 10.1186/s12889-026-26983-4.

## Background

Health and healthcare utilization (HCU) in crime convicted persons (CCPs) are urgent topics in criminology and medicine, with wide-ranging social and political implications, partly due to the high likelihood of death and adverse somatic health outcomes within the group [1, 2]. With an increasing incarcerated population globally, currently over 11 million [3], it is essential to limit adverse somatic outcomes, such as injuries, somatic illness, debilitating disease, and ultimately preterm mortality, in this marginalized group [4].

Several longitudinal studies have documented elevated rates of adverse somatic outcomes and preterm mortality associated with a criminal lifestyle [1, 2, 5–7]. Extensive research conducted across diverse contexts underscores an elevated incidence of adverse somatic outcomes such as somatic illnesses (e.g., cardiovascular disease, infections, and liver complications) and injuries in a variety of crime convicted cohorts from different countries and with diverse characteristics [2, 8–10]. Although the incidence rate of somatic illness inevitably varies throughout an individual’s life course, accumulating evidence demonstrates that CCPs, especially if persistent in criminality, seemingly, face an increased risk of developing such conditions. Using data from the Cambridge Study in Delinquent Development (CSDD), Skinner and colleagues [11] reported that CCPs by the age of 48 were more than twice as likely to suffer somatic illness. However, some data show an inverse relationship between adverse somatic outcomes and persistent offending, a group theorized to have an increased risk of somatic illness and injuries [12, 13], at a young age. Within the CSDD cohort, offending participants between 16 and 18 years old have also shown a lower risk of respiratory tract disease, and less organic illness by the age of 32 [14, 15]. Yet, the primary driver of disease, the biological aging process, seems faster among persistent CCPs [15]. Using extensive data, including validated biomarkers, clinical tests, and self-reports, Langevin and colleagues concluded that persistent offending added 4.3 years of biological aging between the ages 25–45 compared to a group that exhibited low levels of antisocial behavior [15].

The progression of most illnesses is inherently gradual but there are more acute adverse somatic events that immediately affect an individual’s health status, such as injuries. CCPs’ increased propensity for injuries has been widely described, with a severely increased risk of serious and possibly life-threatening outcomes [6, 10, 16]. Despite this, there is little research regarding injuries in persistent CCPs. In the CSDD cohort, Piquero and colleagues found a high propensity for injuries: 27% had suffered injuries between the ages of 27–32 years, and 21% in men between the ages of 43–48 [17]. Additionally, Shepherd and colleagues showed that patterns of injury differed between criminal trajectories, with persistent CCPs being more prone to traffic, industrial, and assault injuries compared to the general population [14].

The increased risk of injuries and somatic disease in the persistent CCP population is likely partly attributable to altered health- and risk behaviors compared to those observed in the general population, as a clear overrepresentation of varying health-threatening behaviors and experiences has been reported in the group [8]. A systemic review concluded that the level of drug use disorders equaled 30%, while alcohol use disorder equaled 24% for males in prison [18]. The incidence of smoking has been shown to be substantially higher compared to the general population and varied around 70–80% in different studies [8]. Additionally, childhood adversities such as abuse or neglect [19–21] prior incarceration [22] unprotected sexual encounters [23], psychiatric morbidity [2, 24], high incidence of psychotic disorders [25], intravenous drug use [18], impulsive high-risk behaviors [26], as well as high-trait aggression [27] merit the group with a higher risk of other adverse somatic outcomes.

Furthermore, mortality rates among CCPs are significantly elevated compared to the general population [1]. The highest mortality rates seem to be found in newly released prisoners [28] as well as individuals released from high-security prisons [29]. Death through external causes such as suicide and intoxications are especially increased among imprisoned youth and CCPs suffering from substance use disorders (SUDs) [7, 28]. An Australian study that analyzed deaths up to one year after prison release showed that almost all the deceased young ex-prisoners died from intoxication or injuries [30]. Notably, CCPs have also shown a high propensity for suicide [31] as well as all-cause mortality [32], with the most persistent CCPs being up to 30 times more likely to have died by the age of 30 compared to non-offending controls [1].

While the literature on adverse somatic outcomes in CCPs is increasing, the literature concerning CCP HCU remains scarce, and there are few longitudinal studies of how persistent offending affects interactions with healthcare providers. Although the Cambridge Study in Delinquent Development found an inverse relationship between offending and inpatient care at age 32 [10, 11, 14], this pattern shifted by age 48, when persistent CCPs demonstrated higher rates of inpatient care and an increased risk of adverse somatic outcomes [11]. Similarly, Rivenbark and colleagues [33] could show an elevated number of emergency visits, prescription fills and injury claims among persistent CCPs from age 26 to age 38 compared to a low conduct problem trajectory. Other work has shown that CCPs released from prison are almost twice as likely to be hospitalized 90 days after their release compared to a non-convicted control group [34]. Allen and colleagues conducted a study in Australia showing that one in five of released prisoners utilized inpatient care within a year of release, with psychiatric disorders and injuries contributing for most of the days of inpatient care [35]. Furthermore, a recent arrest has been shown to predict later emergency and inpatient HCU [36]. Post-release, emergency department visits are frequent, particularly among CCPs with psychiatric disorders and SUD [37, 38]. Other notable risk factors for hospitalization are early-onset antisocial behavior, smoking, and a history of out-of-home-placements [33, 37, 39]. Nevertheless, research on the topic remains unsatisfactory, as Skinner and Farrington [40] recently underscored by pointing to need for further investigations into CCPs’ interaction with health services.

According to the WHO, somatic HCU in groups at a high risk of social exclusion, such as persistent CCPs [41], is not only directly linked to somatic health outcomes, but also reflects social and structural components influencing utilization patterns [42]. Young violent crime convicted persons (VCCPs) is a group at considerable risk of social exclusion, which can emerge as a potent driver of health inequality due to dimensions including unequal patterns of formal rights, cultural norms, and economic disparities and proximal support relationships [42]. Also, studies on the topic have demonstrated that limited health literacy, low educational attainment, as well as cultural and language barriers can deter individuals from seeking appropriate care [43, 44].

This longitudinal study aims to explore how VCCPs with an experience of imprisonment in young adulthood prospectively interact with Swedish somatic healthcare services compared to a matched general population comparison group.

The specific aims of this study were:

1. to describe the prospective HCU, morbidity, injuries, prescription drug use, and mortality from inclusion (March 2010 to July 2012) throughout 2017 in a VCCP cohort in comparison to a matched general population group and.
2. to explore risk factors, including psychiatric, somatic, and social backgrounds, as well as persistent offending and their association with inpatient somatic healthcare utilization in a VCCP cohort.

## Methods

### Participants

The study cohort, the Development of Aggressive Antisocial Behavior Study (DAABS), consisted of young adult (18–25 years of age) male CCPs incarcerated for violent crimes (including contact sexual offenses) across nine correctional facilities in the Western region of the Swedish Prison and Probation Service between March 2010 and July 2012. Among the initial 420 inmates who met the inclusion criteria, 42 persons were excluded: 23 since they could not communicate in Swedish (i.e., required an interpreter), and 19 others for insufficient duration of stay at the current prison. Of the remaining 378 individuals, 109 declined to participate, resulting in a study group of 269 participants. Non-participants showed no differences from the DAABS cohort in terms of median age or type of index crime. Three additional participants were not traceable in national registers, resulting in a final cohort of 266 individuals. The cohort is considered representative of young adult male VCCPs in the Swedish Prison and Probations Service and the cohort’s characteristics, e.g., low levels of education attainment, as well as high levels of adversities, somatic and psychiatric morbidity have been described extensively in several publications [45–48].

A comparison group, consisting of 10,000 individuals, matched by age- and sex was drafted from the general population by the Swedish government agency Statistics Sweden. The follow-up time (person-time) constituted the time from inclusion in the study until the last day of 2017, death, or final registered emigration [45]. The mean follow-up time differed between the groups (*p* < .001). The DAABS cohort average follow-up time was 6.2 years (*SD* = 1.3), while the comparison group average was followed 7.7 years (*SD* = 0.8). The mean age at study end was 28.1 years (*SD* = 2.3) for the DAABS cohort and 28.4 years (*SD* = 2.0) for the comparison group.

### Procedure

At baseline, extensive assessments of the DAABS participants were conducted by licensed clinical psychologists following the LEAD (longitudinal, expert, all data) principles [46, 47]. After baseline, the participants were followed in official Swedish registers until 31 December 2017. For the current study, we retrieved information from the National Patient Register (NPR), the Prescribed Drug Register (PDR), and the Swedish Cause of Death Register (CDR). The NPR includes information about all public and private specialist healthcare in Sweden, specialist inpatient care, and appointments to a medical doctor in specialist outpatient care, but no data on primary care. The PDR holds information about all prescribed drugs that have been dispensed from a Swedish pharmacy, including prescriptions from primary care. The CDR register contains data on all deaths of people registered in Sweden. The National Board of Health and Welfare oversees the NPR, PDR, and CDR.

### Prospective outcomes

#### Adverse health outcomes

We retrieved prospective count data regarding out- and inpatient HCU from the NPR. When an outpatient care visit was followed by an inpatient visit in the same hospital, resulting in the same diagnoses on the same day, we counted only the inpatient visit. Data on somatic morbidity, all-cause, and cause-specific injuries was also collected from the NPR. The NPR has been validated by the National board of Health and Welfare [49] and through external validation [50], showing positive predictive values around 85–95%.in most diagnoses in the NPR.

To describe the occurrence of preventable somatic inpatient HCU in the register, we categorized ICD-codes according to the ambulatory care sensitive conditions (ACSC) system. As Sweden has no validated list on ACSCs data on somatic morbidity, we used the NHS definition [51]. We used a binary variable for any somatic inpatient HCU due to ACSC and then divided ACSC into the three subcategories: chronic, acute, and vaccine-preventable diseases, in close accordance with Satokangas and colleagues [52]. Initially designed to evaluate primary healthcare performance, the ACSC conditions have been proposed as a better tool for identifying sicker individuals [53].

Injuries were categorized according to the ICD classification. For a detailed description of all definitions, see the supplementary material (Table S1).

#### Prescription drugs

Prescription drug use was calculated using data from the PDR and categorized according to the Anatomical Therapeutic Chemical tree resulting in 14 categories [54] (definitions in Table S1). Each prescription’s defined daily dose was cumulated into a continuous variable representing the total prescription drug use during the follow-up period for each category of drugs. Daily defined doses illustrate the assumed maintenance dose of a drug used for its main indication per day [54]. In this study, prescription drug data is primarily used as a proxy for primary HCU and morbidity.

#### Mortality

All-cause and cause-specific mortality was reported (definitions in Table S1). The quality of the Swedish CDR has been estimated in case-reports, investigating individuals who passed away in hospitals, and 77% agreement on the underlying cause of death was found [55]. We opted against incorporating diagnoses categorized as undetermined events into the definitions of suicide or self-harm, in line with the recommendation by Björkenstam and colleagues [56].

### Risk factors

Descriptive data of included risk factors for somatic inpatient healthcare visits are reported in Table S3.

#### Psychiatric background

The Swedish translation of the Structured Clinical Interviews for DSM-IV-Axis I & II Disorders [57] and structured interview protocols according to the Diagnostic and Statistical Manual of Mental Disorders, 4th edition, text revision [58] was used to assess participants’ life-course psychiatric background during baseline interviews. Data on mood disorders, anxiety disorders, psychotic disorders, alcohol and drug use disorders, personality disorders, persistent ADHD, and autism were employed as well as information regarding prior psychiatric inpatient HCU.

#### Somatic background

Risk factors representing somatic background were collected during the extensive baseline assessment. Information regarding traumatic brain injury, chronic somatic illness (variable structure in Table S2), and repeated deliberate self-harm and repeated suicide attempts were employed.

#### Social background

Information on participants’ country of birth, low education level (specifically, those who did not complete high school), and history of foster home or institutional care placements was gathered using a combination of methods. These included semi-structured interviews, self-reports from the participants, and reviews of records at the start of the study and the variables encapsulate the best available information based on these sources.

#### Offending trajectories

Persistence in offending was analyzed using five offending trajectory groups, identified in a previous publication [59], and based on yearly crime rates and patterns over time from age 15 to 29, using group-based trajectory modelling [60]. These trajectory groups were: Low-rate desisters, characterized by few criminal convictions and displaying desistance; Moderate-rate persisters, with early onset of criminal behavior, with a peak at 21, followed by decline; High-rate early-peak persisters, showing consistent high rates of offending, peaking at 16; High-rate late-peak persisters, described by high offending rates peaking at 21; and High-rate inclining persisters, who were the most active trajectory group, with increasing crime rates throughout the study.

### Statistical analysis

To explore the first specific aim, we described HCU, somatic morbidity, prescribed drugs, and mortality in the DAABS cohort compared to the general population comparison group. To provide a comparative overview of this information, we performed unadjusted tests. Thus, for variables involving cumulative count data, i.e., HCU and prescribed drugs, we utilized zero-inflated Poisson regression models [61] and reported incidence rate ratios (IRR). These models incorporated information on follow-up time adjusted for mortality and emigration. Longitudinal research of count data often violates the assumption of independence between observations [62]. To mitigate the risk of false positives, we employed a mixed-model extension estimator of variance in all analyses using zero-inflated Poisson regression. In analysis of HCU, we further adjusted follow-up time by considering the number of somatic inpatient days (DAABS: *M*_*days*_ = 2.4, *SD* = 8.5; Comparison group: *M*_*days*_ = 0.8, *SD* = 5.4). This adjustment accounts for somatic inpatient care not being deemed time-at-risk for concurrent somatic HCU. When analyzing binary information on cumulative incidence, i.e., ACSC and injuries, we used univariable logistic regression and reported odds ratios (OR). For all-cause mortality, we employed a univariable Cox proportional hazard model [60] and reported a hazard ratio (HR).

To investigate the second specific aim, we univariably explored the associations between psychiatric, somatic, and social background as well as offending trajectory group assignment and the frequency of somatic inpatient healthcare visits in the DAABS cohort using zero-inflated Poisson regression. In the DAABS cohort, 152 individuals (57%) did not utilize somatic inpatient care throughout the follow-up period. Additionally, as outliers can have a substantial effect on zero-inflated Poisson distributions, we applied a 90% Winsorization technique [63] to adjust the main outcome in the positive range of the sample. This adjustment resulted in the outcome measure being constrained to a range of 0–9 (*M* = 1.0, *SD* = 1.9) somatic inpatient visits in the DAABS cohort. Lastly, we multivariably explored the direct estimated effect of each category of risk factors on the main outcome (model 1–3). Additionally, we explored the total estimated effect of all risk factor categories, while adding offending trajectory group assignment in model 4. Only variables that exhibited a marked effect (*z*-value > 1.5) on the main outcome in previous multivariable analyses were included in model 4.

All effect sizes were reported with corresponding 95% confidence intervals (CI). Statistical significance was determined at the *p* < .05 level. The statistical software Stata (version 17) was used for all analysis.

## Results

### Adverse health outcomes

Throughout the follow-up period and as reported in Table 1, the DAABS cohort exhibited significantly elevated somatic outpatient and inpatient HCU, compared to the matched general population comparison group.

**Table 1:** Somatic health care utilization and morbidity in VCCPs compared to a general population group

	DAABS (*n* = 266)	Comparison group (*n* = 10,000)	Effect size (CI 95%)
**Health care utilization**	*M (SD)*		**IRR**
Somatic health care visits	8.8 (16.6)	4.4 (6.9)	2.0 (1.6–2.5) *
–Outpatient	7.4 (12.3)	4.1 (6.3)	1.8 (1.5–2.2) *
–Inpatient	1.5 (4.8)	0.3 (1.1)	3.3 (2.2–4.9) *
**Ambulatory care sensitive conditions**	*n* (%)		**OR**
Acute	17 (6)	286 (3)	2.3 (1.4–3.8) *
Chronic	3 (1)	175 (2)	0.6 (0.2–2.0)
Vaccine-preventable	11 (4)	100 (1)	4.3 (2.3–8.1) *
Any	29 (11)	533 (5)	2.2 (1.5–3.2) *
Injuries			
Accident	147 (55)	3,554 (36)	2.2 (1.8–2.9) *
Self-harm	37 (14)	96 (1)	16.7 (11.2–24.9) *
Victim of violence	44 (17)	340 (3)	5.6 (4.0–7.9) *
Undetermined intent	14 (5)	105 (1)	5.2 (3.0–9.3) *
Any	173 (65)	3,735 (37)	3.1 (2.4–4.0) *

*DAABS* Development of Aggressive Antisocial Behaviour Study, *IRR* incidence rate ratio, *OR* odds ratio

*p <.05

During the follow-up, 91% (*n* = 241) of the DAABS cohort and 73% (*n* = 7,345) of the comparison group used outpatient somatic care. Furthermore, 43% (*n* = 114) of the DAABS cohort had used inpatient somatic care, whereas only 18% (*n* = 1,804) used such care in the comparison group.

When looking at different health conditions, the DAABS cohort also exhibited a statistically significant excess in the cumulative incidence of ACSCs (IRR = 2.2 [1.5–3.2]). The most pronounced difference was observed for vaccine-preventable conditions (IRR = 4.3 [2.3–8.1]). Moreover, the DAABS cohort presented an elevated cumulative incidence across all categories of injuries (IRR = 3.1[2.4-4.0]), including accidents, self-harm, victimization, and injuries of undetermined intent. The excess burden of self-harm-related injuries (IRR = 16.7 [11.2–24.9]) was particularly notable, prompting further analysis.

Within the DAABS cohort (*n* = 266), 32 of the 37 participants who utilized healthcare as a result of self-harm had done so on at least one occasion due to self-harm by poisoning, and 13 of these 37 utilized healthcare following self-harm via other causes. By contrast, among the 96 cases in the comparison group (*n* = 10,000) who had utilized healthcare related to self-harm, 64 had done so after poisoning and 37 for other causes with some individuals seeking care for both. However, healthcare use stemming from poison-related accidents was less common than self-harm by poisoning in both groups but occurred more frequently in the DAABS cohort (*n* = 10, 4%) compared with the comparison group (*n* = 58, 0.6%).

### Prescription drug use

We found that the average defined daily dose of prescribed drugs differed on a statistically significant level in several categories between the DAABS cohort and the comparison group. As reported in Table 2, the DAABS cohort showed a heightened incidence rate of prescription drug usage affecting the nervous system and respiratory organs, while the comparison group showed higher incidence rate of prescription drug usage effecting alimentary tract and metabolism, blood and blood forming organs, and skin.

**Table 2:** Dispensed prescription drugs in VCCPs compared to a general population group

	DAABS (*n* = 266)	Comparison group (*n* = 10,000)	Effect size (CI 95%)
**Prescribed drugs, daily defined doses**	*M (SD)*		**IRR**
Alimentary tract and metabolism	39 (109)	103 (644)	0.3 (0.2–0.4) *
Blood and blood forming organs	14 (107)	59 (900)	0.3 (0.1–0.6) *
Cardiovascular system	37 (312)	19 (234)	1.3 (0.5–3.5)
Dermatologicals	4 (28)	8 (70)	0.6 (0.3–0.9) *
Genito-urinary systems and sex hormones	8 (61)	7 (91)	0.9 (0.4–2.1)
System hormonal preparations	11 (91)	18 (190)	0.7 (0.3–1.9)
Anti-infectives for systemic use	27 (117)	22 (65)	1.5 (0.9–2.5)
Antineoplastic and immunomodulating agents	0 (0)	12 (158)	-
Muscolo-skeletal systems	34 (117)	25 (121)	1.4 (1.0–2.1)
Nervous system	897 (2441)	287 (1172)	2.3 (1.7–3.2) *
Antiparasitic products, insecticides, and repellents	0 (1)	1 (18)	-
Respiratory system	202 (632)	109 (436)	1.7 (1.2–2.4) *
Sensory organs	0 (0)	1 (51)	-
Various	0 (0)	0 (6)	-

*DAABS* Development of Aggressive Antisocial Behavior Study, *IRR* incidence rate ratio

*p <.05

### Mortality

In the DAABS cohort, 7% (*n* = 18) were deceased throughout at the end of follow-up, contrasting notably with the age- and gender matched comparison group, in which less than 1% (*n* = 54) had died (Table 3). The relative risk of all-cause death was statistically significantly increased and profound in the DAABS cohort (HR 16.1 [9.4–27.8]). The leading cause of death was accidents in both groups, although the proportion of the group who had died due to accidents were markedly increased in the DAABS cohort. In addition, suicide represented a more common cause of death in the DAABS cohort compared to the comparison group. Notably, no deaths resulting from violence occurred in the comparison group, while three participants within the DAABS cohort had died as victims of violence. Death due to somatic disease was rare in both groups.

**Table 3:** All-cause and cause-specific mortality in VCCPs compared to a general population group

	DAABS (*n* = 266)	Comparison group (*n* = 10,000)
Mortality	*n* (%)	
Total	18 (7)	54 (0.5)
Somatic disease	1 (0.4)	15 (0.2)
Accident	10 (4)	23 (0.2)
Suicide	3 (1)	13 (0.1)
Victim of violence	3 (1)	0 (0)
Undetermined intent	1 (0.4)	3 (0.03)

*DAABS* Development of Aggressive Antisocial Behavior Study

* *p* <.05

### Exploring risk factors associated with somatic inpatient HCU

Table 4 presents explorative analyzes of the univariable association between each analyzed risk factor on total prospective somatic inpatient HCU in the DAABS cohort during the follow-up time. Anxiety disorders, low educational attainment, and history of out-of-home placement in a foster home were found to be univariably associated with the outcome on a statistically significant level. Additionally, assignment to three out of the four previously identified persistent offending trajectory groups was associated with increased incidence of somatic inpatient HCU.

**Table 4:** Univariable regression models of risk factors on total somatic inpatient health care utilization in VCCPs

	IRR (CI 95%)
Psychiatric background	
Mood disorders	1.3 (0.8–2.0)
Anxiety disorders	1.8 (1.1–3.3) *
Psychotic disorders	1.4 (0.7–2.8)
Alcohol use disorder	1.1 (0.7–1.6)
Drug use disorders	1.1 (0.5–2.5)
Personality disorders	1.0 (0.6–1.6)
ADHD, persistent	1.2 (0.8–1.9)
Autism	1.1 (0.6–2.3)
Prior psychiatric inpatient care	1.5 (1.0–2.4)
Somatic background	
Traumatic brain injury	0.9 (0.5–1.5)
Chronic somatic illness	0.9 (0.5–1.3)
Repeated deliberate self-harm	0.9 (0.5–1.7)
Repeated suicide attempts	1.2 (0.6–2.1)
Social background	
Immigrant status	0.8 (0.5–1.3)
Low educational attainment	0.6 (0.4–1.0) *
Placement, foster home	1.8 (1.2–2.8) *
Placement, institutional care	1.2 (0.8–1.8)
Repeated domestic violence, victim	1.5 (1.0–2.3)
Trajectory group	
Low rate desisters	(reference)
Moderate rate persisters	3.2 (1.7–5.8) *
High-rate late-peak persisters	3.5 (1.8–6.7) *
High-rate early peak persisters	1.8 (0.9–3.3)
High-rate inclining persisters	5.7 (3.1–10.6) *

*IRR* incidence rate ratio

* *p* <.05

Subsequently, we performed a series of multivariable regression analyses (see Table 5). In turn, analyzing each category, i.e., psychiatric, somatic, and social background, of risk factors’ associations with total somatic inpatient HCU. In model 1 and 2, which explored the associations between psychiatric and somatic background, respectively, on total somatic inpatient visits, no association remained statistically significant. In model 3, exploring the association between social background and the outcome, low educational attainment was negatively associated, while a history of out-of-home placement in a foster home was positively associated with total somatic inpatient healthcare visits.

**Table 5:** Multivariable regression models of risk factors on total somatic inpatient health care utilization in VCCPs

Risk factors	Model 1	Model 2	Model 3	Model 4
	IRR (95% CI)			
Psychiatric background				
Mood disorders	0.8 (0.4–1.4)			
Anxiety disorders	1.9 (0.8–4.6)			
Psychotic disorders	1.3 (0.5–3.3)			
Alcohol use disorder	0.9 (0.5–1.6)			
Drug use disorders	1.1 (0.4–2.7)			
Personality disorders	1.0 (0.5–1.8)			
ADHD, persistent	1.1 (0.6–2.1)			
Autism	1.2 (0.6–2.5)			
Prior psychiatric inpatient care	1.3 (0.7–2.2)			
Somatic background				
Traumatic brain injury		0.9 (0.6–1.4)		
Chronic somatic illness		0.8 (0.5–1.3)		
Repeated deliberate self-harm		1.0 (0.5–1.9)		
Repeated suicide attempts		1.2 (0.7–2.1)		
Social background				
Immigrant status			0.9 (0.6–1.3)	
Low educational attainment			0.5 (0.3–0.8) *	0.5 (0.3–0.7) *
Placement, foster home			1.9 (1.1–3.2) *	1.8 (1.2–2.6) *
Placement, institutional care			1.1 (0.6–1.7)	
Repeated domestic violence, victim			1.3 (0.9–2.0)	
Trajectory group				
Low-rate desisters				(reference)
Moderate-rate persisters				2.9 (1.6–5.2) *
High-rate late-peak persisters				4.1 (2.3–7.4) *
High-rate early peak persisters				2.0 (1.1–3.8) *
High-rate inclining persisters				5.9 (3.0–11.7) *

*IRR* incidence rate ratio

* *p* <.05

Lastly, in model 4, accounting for all categories of previously analyzed risk factors, using risk factors that in previous models indicated a marked effect on the outcome, as well as offending trajectories, low educational attainment (IRR 0.5 [0.3–0.7]) and a history of out-of-home placement in a foster home (IRR 1.8 [1.2–2.6]) remained associated with prospective total somatic inpatient visits. In the same multivariable model, assignment to a persistent offending trajectory was significantly associated with a higher incidence of somatic inpatient HCU compared to desisters (IRR 2.0 [1.1–3.8] – 5.9 [3.0–11.7]).

## Discussion

To date, there is a lack of studies investigating adverse health outcomes and HCU among persistent CCPs and individuals with a history of violent convictions. This study aimed at alleviating this knowledge gap by exploring longitudinal prospective adverse health outcomes and mortality, as well as risk factors associated with somatic inpatient HCU in VCCPs with a history of imprisonment in young adulthood (the DAABS cohort) over a period of more than six years compared to a matched general population comparison group.

Our main finding was that the DAABS cohort showed increased rates of both somatic inpatient and outpatient HCU compared to a matched comparison group. Furthermore, we found that assignment to a persistent offending trajectory group was associated with elevated somatic inpatient HCU.

The results of an increased HCU are consistent with previous research on HCU among people who experience incarceration [11, 64–66], which similarly reported increased overall utilization.

Our findings regarding ACSCs, injuries, prescription drug use, and mortality. The elevated levels of HCU due to ACSCs which we found in the DAABS cohort, aligns with earlier studies showing that individuals with a history of imprisonment are more likely to utilize somatic healthcare for acute conditions compared with the general population [67]. Another plausible contributing factor is healthcare inequality and a reluctance to seek proper care [68, 69]. Even within low- or free-cost healthcare systems, such as the Swedish healthcare system, inequalities arise consequently affecting individual health, preventive medication, and HCU [42]. The elevated incidence of ACSCs in the DAABS cohort may indicate lower utilization of, or barriers to, primary care services, as ACSCs are generally considered preventable through timely and effective outpatient management.

Interestingly, the DAABS cohort demonstrated a markedly elevated risk of somatic inpatient healthcare utilization due to vaccine-preventable ACSCs. Although the Swedish childhood vaccination program has achieved high coverage, with an uptake of 97% [70], these findings may suggest that members of the cohort grew up in marginalized families where deviant HCU included denying them basic pediatric healthcare as children, but additional investigations into vaccination coverage and infectious diseases are needed to fully understand the heightened levels of vaccine-preventable disease within this group. Second, the cumulative incidence of an injury diagnosis was significantly elevated for the DAABS cohort during the follow-up compared to the comparison group. These results are in line with prior studies of literature on CCPs’ high propensity to injuries [11–13, 71]. Third, and also consistent with previous research [19, 48], the DAABS cohort exhibited an increased risk of prescription drug use affecting the nervous system. This finding aligns with increased rates of nervous system and psychiatric morbidity among CCPs. Notably, several other categories of medication were more frequently prescribed in the comparison group, which may suggest a higher threshold for somatic HCU among CCPs or a greater reluctance among clinicians to prescribe certain medications to this population. Lastly, the DAABS cohort exhibited a severely elevated risk of mortality, in accordance with previous literature on persistent CCPs [1]. The high mortality rates, likely attributed to SUD [18], a criminal lifestyle [1], aggression [27], and other health-threatening behaviors as well as psychiatric morbidity [25] may also indicate a reluctance among the DAABS participants to seek healthcare, and thus influence the register-based HCU outcomes in the cohort.

This study also examined risk factors for somatic inpatient HCU. In the univariable model, no psychiatric or somatic background variables were significantly associated with somatic inpatient HCU, possibly due to the homogeneity of the analyzed risk factors in the cohort. However, low educational attainment decreased the incidence, while foster home placement and persistent crime trajectories independently increased the incidence of somatic inpatient HCU on a statistically significant level. Sariaslan and colleagues [39] have previously shown an increased risk of adverse somatic health outcomes, e.g., injuries and intoxications, in adulthood among children placed in out-of-home-care, especially among individuals also convicted of a violent crime. In accordance, our results might suggest a higher need for somatic inpatient HCU in previously foster home placed young VCCPs. However, another plausible explanation, drawing from the WHO framework regarding HCU, inequality, and social exclusion [42], one speculative factor may involve that foster home, as compared to institutional, placements can contribute to improved health literacy as well as access to caring and attentive caregivers who encourage appropriate medical visits. Furthermore, it should be noted that the DAABS cohort consists of CCPs, a group in which exposure to childhood abuse and other adverse experiences is more common than in the general population. Prior research shows that early-life adversities are strongly associated with increased HCU and poorer health outcomes later in life [20, 21]. For example, in a study of CCPs in a prison population, high levels of adverse childhood experiences were strongly linked with self-harm behavior, mental health diagnoses, and other negative health outcomes.

Our results did also show a highly elevated incidence of somatic inpatient use for persistent CCPs. This finding could be partly explained by traits such as high impulsivity, aggression, a risk-taking lifestyle, or a higher incidence of childhood adversities increasing the possibility of injuries, somatic disease [11–14, 17, 22].

### Limitations

The first limitation of our study is the nature of register-based data on adverse somatic outcomes being dependent on HCU. The study reported on HCU as it naturally occurred and did not explore the timing of HCU or its relation to other time-covarying events, such as release from prison or reimprisonment.

The second limitation is the difficulties of comparing our results to literature with a similar methodology, group division and aims [40]. While few register-based studies on HCU among VCCPs exist, comparisons are feasible within a broader spectrum of studies involving CCPs in general and diverse methodologies.

The third limitation involves conducting within-group analyses within the DAABS cohort. Although diverse in certain aspects, this cohort shares homogeneity in terms of risk factors like psychiatric morbidity, adverse upbringing, and aggression, potentially influencing the regression analyses.

The fourth limitation is that the DAABS cohort consists exclusively of men, rendering generalization for female CCPs non-viable.

The fifth limitation is the lack of data on victimization. Since victimization is a well-known risk factor for increased healthcare use (HCU), this missing information limits our ability to fully understand the pathways leading to HCU among VCCPs. Including victimization data would have added valuable context, helping to better explain the relationship between violence and healthcare needs in this group.

A sixth limitation is the small cell sizes in some analyses, which may reduce precision and limit the generalizability of the findings. Larger studies are needed to validate these results.

## Conclusions

In this study, VCCPs with a history of imprisonment in young adulthood exhibited an atypical somatic HCU pattern compared to a general population, characterized by a high cumulative incidence of ACSCs and increased incidence of both somatic inpatient and outpatient care during the prospective follow-up period. The incidence of inpatient somatic HCU was particularly elevated among individuals in the persistent trajectory groups. Low educational attainment decreased the incidence of somatic inpatient HCU, while foster home placement increased the incidence, which might be attributable to the positive effects of health literacy promoted by such an experience. The atypical nature of HCU, reflected in the disproportionately high prevalence of ACSCs, suggests barriers to timely and appropriate primary HCU. This has clinical implications in terms of how appropriate care is secured for exposed groups. Early and improved identification of at-risk individuals and outreach to exposed communities are important to attain a higher level of equality within the healthcare systems. Further, VCCPs suffered a severely elevated risk of mortality even among this young cohort. Thus, making early intervention necessary to prevent somatic adverse events and preterm mortality within the group. Developing psychoeducation and health literacy, in settings such as prison and out-of-prison programs, regarding when, where and how to utilize healthcare can be one step forward.

## Supplementary Information

Supplementary Material 1.

## Data Availability

The datasets generated and/or analyzed during the current study are not publicly available due to the participants’ privacy rights but are available from the corresponding author upon reasonable request.

## Abbreviations

CDR: Cause of Death Register
CI: Confidence interval
CSDD: Cambridge Study in Delinquent Development
HCU: Healthcare utilization
HR: Hazard ratio
IRR: Incidence rate ratio
NPR: National Patient Register
OR: Odds ratio
PDR: Prescribed Drug Register
SUD: Substance use disorders
