# Supplementary material for: Adverse health outcomes in violent crime convicted persons: risk factors for somatic inpatient healthcare utilization
Source: BMC Public Health. 2026 Mar 24;26:1167. doi: 10.1186/s12889-026-26983-4 (PMC13064182; doi:10.1186/s12889-026-26983-4)
Supplement: Supplementary file 1 — Supplementary Material 1. [file 12889_2026_26983_MOESM1_ESM.docx]

**Table S1. Definitions of morbidity, injuries, prescribed drugs, and mortality.**

| **Ambulatory care sensitive conditions** | **ICD-10 codes** |
| --- | --- |
| Acute | Primary diagnosis only:  L03/L04 L080 L088 L88 L980  G40/G41 O15 R56  E86 K522 K528 K529  A690 K02/K06 K08 K098 K099 K12/K13  N10/N12 N136  N70 N73/N74  K250/K252 K254/K256 K260/K262 K264/K266 K270/K272 K274/K276 K280/K282 K284/K286  H66/H67 J02/J03 J06 J312  In any diagnosis field:  R02 |
| Chronic | Primary diagnosis only:  I20 I240 I248 I249  J45/J46  J41/J44 J47  I110 I50 J81  E100/E108 E110/E118 E120/E128 E130/E138 E140/E148  I10 I119  D501/D509  E40/E43 E550 E643  Only when primary diagnosis in combination with J41/J44 J47 in any diagnosis field:  J20 |
| Vaccine-preventable | In any diagnosis field:  A35/A37 A80 B05/B06 B161 B169 B180 B181 B26 G000 M014  In any diagnosis field and not D57 in any other diagnosis field:  J09/J11 J13/J14 J153 J154 J157 J159 J168 J181 J188 J189 |
| **Injuries** |  |
| Accident | V00/X59 |
| Self-harm | X60/X84 |
| Victim of violence | X85/Y09 |
| Undetermined intent | Y10/Y34 |
| **Prescribed drugs** | **ATC prefix** |
| Alimentary tract and metabolism | A |
| Blood and blood forming organs | B |
| Cardiovascular system | C |
| Dermatologicals | D |
| Gentio urinary systems and sex hormones | G |
| System hormonal preparations | H |
| Antiinfectives for systemic use | J |
| Antineoplastic and immunomodulating agents | L |
| Muscolo-skeletal systems | M |
| Nervous system | N |
| Antiparasitic products, insecticides and repellents | P |
| Respiratory system | R |
| Sensory organs | S |
| Various | V |
| **Mortality** | **ICD-10 codes** |
| Somatic disease | A00/R99 |
| Accident | V00/X59 |
| Suicide | X60/X84 |
| Victim of violence | X85/Y09 |
| Undetermined intent | Y10/Y34 |

**Table S2. Variable structure of chronic somatic illnesses.**

| **Chronic somatic illness** | ***n* (%)** |
| --- | --- |
| Epilepsy | 3 (1) |
| Diabetes | 1 (0.4) |
| Thyroid disease | 0 (0) |
| Other endocrinopathies | 0 (0) |
| Migraine | 45 (17) |
| Other neurological disorders | 1 (0.4) |
| Asthma | 42 (16) |
| Autoimmune diseases | 1 (0.4) |
| Serious genetic diseases | 1 (0.4) |
| Cancer | 0 (0) |
| Serious dermatological diseases | 1 (0.4) |
| Total | 86 (32) |

**Table S3. Descriptive data of risk factors for somatic inpatient healthcare visits.**

|  | *N* | *n* (%) |
| --- | --- | --- |
| **Psychiatric background** |  |  |
| Mood disorders | 264 | 141 (53) |
| Anxiety disorders | 264 | 134 (51) |
| Psychotic disorders | 265 | 20 (8) |
| Alcohol use disorder | 266 | 128 (48) |
| Drug use disorders | 266 | 208 (78) |
| Personality disorders | 260 | 173 (67) |
| ADHD, persistent | 263 | 112 (43) |
| Autism | 265 | 26 (10) |
| Psychiatric care, inpatient | 266 | 76 (29) |
|  |  |  |
| **Somatic background** |  |  |
| Traumatic brain injury | 258 | 198 (77) |
| Chronic somatic illness | 266 | 86 (32) |
| Repeated deliberate self-harm | 266 | 32 (12) |
| Repeated suicide attempts | 266 | 26 (10) |
|  |  |  |
| **Social background** |  |  |
| Immigrant status | 266 | 117 (44) |
| Low educational attainment | 265 | 204 (77) |
| Placement, foster home | 266 | 68 (26) |
| Placement, institutional care | 265 | 102 (38) |
| Repeated domestic violence, victim | 265 | 40 (107) |
|  |  |  |
| **Trajectory group** |  |  |
| Low-rate desisters | 266 | 83 (31) |
| Moderate-rate persisters | 266 | 91 (34) |
| High-rate late-peak persisters | 266 | 39 (15) |
| High-rate early-peak persisters | 266 | 36 (14) |
| High-rate inclining persisters | 266 | 17 (6) |
